# Uveal melanoma with a GNA11/GNAQ mutation secretes VEGF for systemic spread

**DOI:** 10.1038/s41392-025-02144-8

**Published:** 2025-02-10

**Authors:** Nguyễn Thị Thanh Nhàn, Sanjay Ganesh, Daniel E. Maidana, Michael J. Heiferman, Kaori H. Yamada

**Affiliations:** 1 Department of Pharmacology & Regenerative Medicine, University of Illinois College of Medicine, Chicago, IL 60612 USA; 2 Department of Ophthalmology & Visual Sciences, University of Illinois College of Medicine, Chicago, IL 60612 USA

**Keywords:** Eye cancer, Metastasis

**Dear Editor**

Uveal melanoma (UM) is a deadly ocular cancer highly metastasizing to the liver.^1^ The vascular barrier in the eyes is critical in preventing UM metastasis. However, the mechanism by which melanoma escapes from the eyes is mainly unknown. Metastatic UM tumors are highly vascularized with leaky blood vessels due to excessive vascular endothelial growth factor (VEGF) production.^2^ Here, we discovered that UM cells with commonly found mutations (GNA11^Q209L^ or GNAQ^Q209L^) rely on VEGF to permeabilize the endothelial barrier and promote UM cell migration across the endothelium.

First, we investigated the endothelial permeability using a gelatin-trapping assay. We cultured human retina endothelial cells (hREC) to form a monolayer on top of the biotinylated gelatin and stimulated them with conditioned media (CM) from UM culture, UM/EC co-culture, hREC culture (negative control), or recombinant VEGF-A (positive control) for 4 hours. We tested MP41 (GNA11^Q209L^) (ATCC), 92.1 (GNAQ^Q209L^, EIF1AX^G6D^) (Sigma), and Mel202 (GNAQ^Q209L/R210K^, CDKN2A^L65R^, SF3B1^R625G^) (Sigma) as human UM cell lines. Note: UM are usually initiated by a mutation in GNAQ or GNA11, thus, almost all UM carry a mutation in GNAQ or GNA11.^1^ The leakage areas were visualized by Alexa555-labeled streptavidin, which is trapped in the biotinylated gelatin at the site of the barrier opening. Co-staining with VE-Cadherin indicates the endothelial barrier. Remarkably, the leakage areas of hRECs were significantly increased upon treatment with CM from all UM cultures and all UM/EC co-cultures or VEGF (positive control) (Fig. 1a). Given that VEGF is highly expressed in the eyes of UM patients,^2^ we tested the effect of VEGF in CM by neutralizing VEGF with Aflibercept. Strikingly, the effect of all CM was notably attenuated by anti-VEGF, indicating a restorative impact (Fig. 1a).

**Fig. 1:**
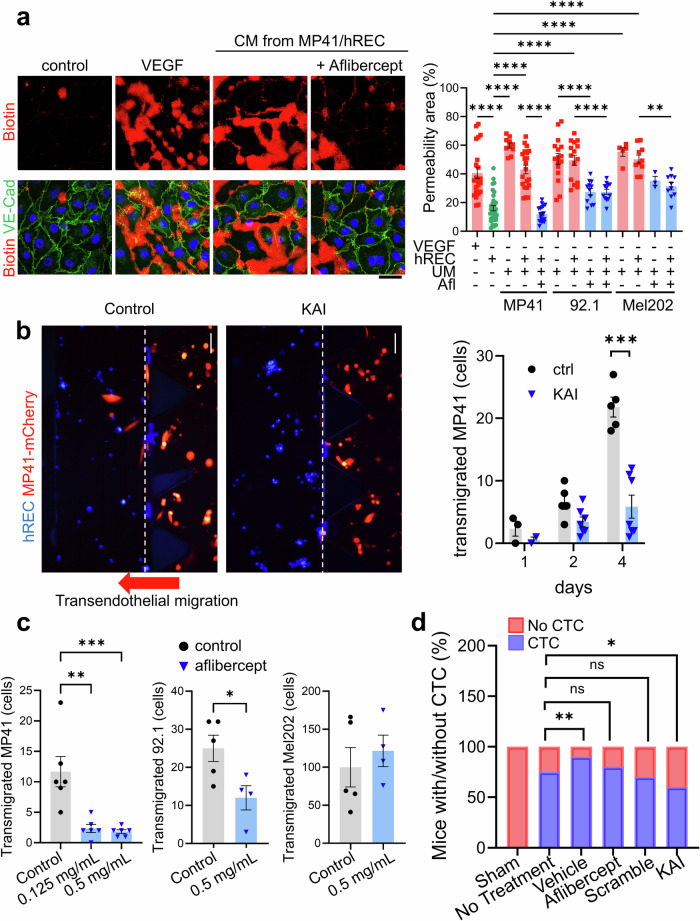
UM induces endothelial permeability to facilitate transmigration by secreting VEGF. **a** Gelatin trapping assay showing endothelial permeability by UM-derived VEGF. Leakage of hRECs monolayer was detected by Alexa555-streptavidin (red) bound to biotinylated gelatin under endothelial monolayer. Counterstaining with VE-Cadherin (VE-Cad, green) indicates endothelial barriers. Conditioned media (CM) from UM (MP41, 92.1, or Mel202) and UM/EC co-culture (MP41, 92.1, or Mel202 together with hREC) induced endothelial permeability, whereas CM from hREC culture (control) did not. Anti-VEGF (Aflibercept 2.5 mg/mL) inhibited the effect of the CM from UM culture and UM/EC co-culture, suggesting the contribution of VEGF in the CM to the permeability. As a positive control, VEGF (50 ng/mL) also induced endothelial permeability. The leakage area (%) was quantified based on Alexa555-streptavidin staining and shown in graph as mean ± SE. *N* = 22, 33, 10, 21, 18, 16, 15, 12, 12, 5, 10, 3, 10 in VEGF, control (CM from hREC), CM from MP41, MP41/EC, MP41/EC + Aflibercept, 92.1, 92.1/EC, 92.1 + Aflibercept, 92.1/EC + Aflibercept, Mel202, Mel202/EC, Mel202 + Aflibercept, Mel202/EC + Aflibercept, respectively. **b** The inhibitory effect of KAI on the transmigration of MP41 (mCherry, red) through the hREC barrier (blue). The number of cancer cells in the left channel (transmigrated cells) was counted daily and shown in the graph. *N* = 5 and 7 for scrambled control peptide (ctrl) and KAI, respectively. *** indicates *p* < 0.001. **c** The dose-dependent inhibitory effect of anti-VEGF (Aflibercept, 0.125 mg/mL and 0.5 mg/mL) on MP41-mCherry transendothelial migration in the organ-on-a-chip. *N* = 5, 6, 6 for control, Aflibercept 0.125 mg/mL, and 0.5 mg/mL, respectively. **p < 0.01, ***p < 0.001 One-way ANOVA, and post hoc comparison. The inhibitory effect of anti-VEGF (Aflibercept 0.5 mg/mL) on 92.1 transendothelial migration and no effect of anti-VEGF (Aflibercept 0.5 mg/mL) on Mel202 transendothelial migration were examined by Student *t*-test. * indicates *p* < 0.05. *N* = 5, 4, 5, 4, for 92.1 control, 92.1 + Aflibercept, Mel202 control, and Mel202 + Aflibercept, respectively. **d** The effect of daily treatment with KAI eyedrop on intravasation of UM cells into circulation. After suprachoroidal injection of MP41 cells in the right eyes of SCID mice, mice were either untreated (no treatment), received an intravitreal injection of Aflibercept (2 µg) or vehicle, received daily treatment of KAI eyedrop (5 µg) or scrambled control eyedrop (5 µg). DNA was isolated from mouse blood, and circulating tumor cells (CTC) in the blood were detected by qPCR using primers for human Chromosome 17. The sham group (tumor-free) served as control. The number of mice with CTC and without CTC (below the levels in the sham group) were counted and shown in the graph. *N* = 5, 4, 10, 10, 10, 10 for sham, no-treatment, Aflibercept, vehicle, KAI, and scrambled control, respectively. *p < 0.05

To test UM cell transmigration, we used organ-on-a chip, where UM cells were mixed with collagen gels and introduced in the middle lane, and blue-stained hRECs were seeded to form the barrier on the surface of collagen gel. In control, MP41-mCherry effectively transmigrated through the endothelial barrier (Fig. 1b, c). The effect of neutralizing VEGF was tested by mixing Aflibercept (0.125 mg/mL or 0.5 mg/mL) with UM cells and collagen gel in the middle lane. The transmigration of MP41 and 92.1 was inhibited by anti-VEGF (Aflibercept) (Fig. 1c), indicating that these UM cells rely on VEGF to transmigrate through the endothelial barrier. Anti-VEGF failed to impede the transmigration of Mel202 with multiple mutations (Fig. 1c), indicating the existence of VEGF-independent pathways.

VEGF induces the endothelial barrier opening via binding to its receptor VEGFR2. For such VEGF/VEGFR2 interaction, trafficking of VEGFR2 to the endothelial cell surface by the kinesin family molecular motor KIF13B is critical.^3^ Given the success of peptide-based strategies owing to their specificity and applicability, we introduce a novel peptide named KAI. Specifically targeting VEGFR2 trafficking, KAI effectively inhibits tumor angiogenesis and extravasation of skin melanoma in mice.^3^ We tested the effect of KAI by adding KAI or control peptide (10 µM) to the left lane where hREC formed a barrier. Inhibition of VEGFR2 trafficking by KAI effectively impeded the transendothelial migration of MP41 compared with the scrambled control peptide treatment (Fig. 1b).

To further investigate the potential inhibitory effects of KAI on UM metastasis, we conducted a comprehensive experiment using an orthotopic mouse model. As the MP41 responded well to anti-VEGF strategies, we focused on MP41 for in vivo study. We expressed mCherry and Nluc (luciferase) in MP41 (MP41-mCherry-Nluc) and injected them into the suprachoroidal space of the right eyes of severe combined immune deficient (SCID) mice. Tumor-bearing mice were divided into five groups to ensure a similar distribution of tumor burden across each treatment group and control group. The five groups are 1) no treatment group, 2) an intravitreal injection of Aflibercept (2 µg/eye) one week post UM implantation, 3) an intravitreal injection of vehicle, 4) daily eyedrop treatment of KAI (5 µg/eye), and 5) daily eyedrop treatment of control peptide (5 µg/eye). The exponential growth of implanted MP41 was monitored by bioluminescence, however, there was no significant difference between groups (data not shown). The primary endpoint was the presence of circulating tumor cells (CTCs), measured at 8 weeks post-UM implantation. Because most of the UM metastasis was found in the liver (91%),^1^ where capillaries are fenestrated, CTCs can easily extravasate through liver blood vessels to metastasize there. CTCs were detected by testing blood from mice for quantitative real-time PCR (qPCR) with primers specific to human chromosome 17 (Cr17)^4^ and mouse GAPDH (mGAPDH). KAI treatment reduced the number of mice with CTCs compared to the no-treatment group (Fig. 1d), suggesting the potential effect of KAI in reducing UM metastasis in mice.

In summary, our study investigated the mechanisms by which UM cells disrupt the endothelial barrier and promote metastasis. We demonstrated that UM cells secreted VEGF to induce endothelial permeability, facilitating UM cell transmigration across the endothelium. The UM cell lines with a mutation in GNA11^Q209L^ or GNAQ^Q209L^ with EIF1AX^G6D^ responded well to anti-VEGF treatment. EIF1AX^G6D^ is generally not correlated to metastatic behavior, unlike SF3B1 mutation in Mel202. Transendothelial migration of Mel202 (GNAQ^Q209L/R210K^, CDKN2A^L65R^, SF3B1^R625G^) was not inhibited by anti-VEGF treatment, suggesting reliance on other pathways. Studying how UM cells affect ECs to facilitate UM transendothelial migration by analyzing gene expression profiles in EC co-cultured with UM cells would be beneficial in defining VEGF-dependent and independent mechanisms and developing combination therapies.

Systemic Aflibercept treatment benefits patients with metastatic UM in a randomized phase 2 study.^5^ Systemic KAI treatment also inhibits the extravasation of circulating melanoma in mice.^3^ Inhibition of VEGF can affect many aspects of tumor growth and metastasis, including angiogenesis and vascular leakage in primary tumors, primary tumor growth, intravasation, extravasation, and colonization of cancer cells in distal organs. Thus, the mechanisms of beneficial effects and any unexpected paradoxical effects need to be investigated in proper animal models. This study uses in vitro models and in vivo suprachoroidal injection of UM cells to focus on the intravasation of UM cells from the eyes through vasculature in the eyes. Using these systems, we showed the inhibitory effect of KAI on UM cells escaping into circulation. We believe this study provides important information for understanding the mechanisms of UM metastasis.

## Supplementary information

Supplemental material

## Data Availability

The authors declare that the data supporting the findings of this study are available within the paper and its Supplementary materials. Raw data and further information are available from the corresponding author on request. The requests for resources and reagents should be directed to and will be fulfilled by the Lead Contact, Kaori Yamada (horiguch@uic.edu).
