# Supplementary material for: Uveal melanoma with a GNA11/GNAQ mutation secretes VEGF for systemic spread
Source: Signal Transduct Target Ther. 2025 Feb 10;10:51. doi: 10.1038/s41392-025-02144-8 (PMC11808083; doi:10.1038/s41392-025-02144-8)
Supplement: Supplementary file 1 — Supplemental material [file 41392_2025_2144_MOESM1_ESM.docx]

Supplementary Materials for

Uveal melanoma with a GNA11/GNAQ mutation secretes VEGF for systemic spread

Nguyễn Thị Thanh Nhàn^1^, Sanjay Ganesh^1^, Daniel E. Maidana^2^, Michael J. Heiferman^2^, Kaori H. Yamada^1, 2, *^

Correspondence to: Kaori H. Yamada horiguch@uic.edu

**This PDF file includes:**

Materials and Methods

Materials and Methods

Reagents

Rabbit antibody against VE-Cadherin was from Cell Signaling (Danvers, MA, USA). Secondary antibodies were Alexa 488 anti-rabbit antibody and Alexa 555-streptavidin (Invitrogen, Waltham, MA, USA). Recombinant human VEGF-A^165^ was purchased from Peprotech (Cranbury, NJ, USA). Eylea (Aflibercept) was from Regeneron Pharmaceuticals, Inc. (Tarrytown, NY, USA).

Cell culture

As ocular endothelial cells, we used human retina endothelial cells (hRECs) because of the unavailability of choroidal endothelial cells. hRECs (Cell Systems, Kirkland, WA, USA) were maintained with EGM2 MV (Lonza Group, Basel, Switzerland). Uveal melanoma cells MP41 (GNA11^Q209L^) (ATCC, Manassas, Virginia, USA), 92.1 (GNAQ^Q209L^, EIF1AX^G6D^) (Sigma St. Louis, MO, USA), and Mel202 (GNAQ^Q209L/R210K^, CDKN2A^L65R^, SF3B1^R625G^) (Sigma) were maintained with RPMI-1640 Medium (Sigma) supplemented with 10% FBS (Invitrogen).

Peptides

KAI peptide was designed from a sequence of human KIF13B based on the minimum binding site for VEGFR2 (PMID: 27863212). Scrambled control peptide was developed as described previously (PMID: 36928770). Both peptides were synthesized with biotin-tag by a custom synthesized service (Thermo Fisher Scientific, Waltham, MA, USA).

Gelatin trapping assay

5 x 10^5^ hRECs were cultured in 6 cm dishes, and 5 x 10^5^ UM cells (MP41, 92.1, and Mel202) were added to hREC culture one day after plating of hREC. Single cultures of UM cells were also cultured in 6 cm dishes. Both monoculture and co-cultures were incubated in serum-reduced endothelial media (EBM2, supplemented with 2% FBS, GA2000, hydrocortisone, and ascorbic acid) for 2~3 days. Conditioned media (CM) were collected and passed through 0.45 µm syringe filters and stored at 4^o^C. Gelatin trapping assay was performed as described previously (PMID: 23212101). 8-well Nunc Lab Tek II Chamber slides (Sigma) were coated with 0.25 mg/mL biotinylated gelatin in 0.1 M bicarbonate buffer (pH 8.3) at 4°C overnight. The 8-well slides were washed twice with PBS (pH 7.4, 200 µL per well), and hRECs were plated to form a confluent monolayer. Subsequently, they underwent a 4-hour treatment with VEGF, conditioned media (CM) from UM culture, UM/EC co-culture, or CM supplemented with Aflibercept at a concentration of 2.5 mg/mL. Following the treatment, cells were washed with PBS and incubated with Streptavidin, Alexa Fluor™ 555 conjugate for 1 minute, washed once with PBS, and fixed with 4% PFA (Electron Microscopy Sciences, Hatfield, PA, USA) for 20 min at room temperature. Cells were permeabilized with 0.1% triton-X100 and incubated with 4% BSA and 10% normal donkey serum (Jackson Immunoresearch) in PBS 0.1% Tween 20. Cells were incubated overnight with specific primary antibodies, followed by fluorescently labeled secondary antibodies with Alexa-488 (Invitrogen). Stained cells were observed under a Zeiss confocal LSM 880 META with a 40× oil immersion objective lens. The confocal images were all obtained in the same microscope settings, such as gain and scanning time.

Organ-on-a-chip assay

MP41 cells were transduced with lentivirus encoding mCherry (MP41-mCherry). 92.1 and Mel202 were stained with CellBrite red (Biotum, Fremont, CA, USA) for 20 min at 37^o^C in the dark. 0.8 x 10^6^ cells/mL MP41 mCherry cells or red-stained 92.1 and Mel202 cells were mixed with 3D ECM (2.5 mg/mL Collagen type I, 1xM99 medium, 0.02N NaOH) and Aflibercept (0, 0.125, or 0.5 mg/mL). 8 µL UM cells mixture was loaded into the middle lane of the AIM Biotech 3D cell culture chip (MatTek Life Sciences, Ashland, MA, USA). The chips were incubated for 2-3 hours at 37^o^C, 5% CO_2,_ to allow the polymerization. 10^6^ cells/mL hREC in EGM2 MV were labeled by CellBrite Blue Cell Labeling Solution (100:1 v/v) (Biotum, Fremont, CA, USA), for 20 min at 37^o^C in the dark. hRECs were washed 3 times with EGM2 MV. hRECs were resuspended in 5 x 10^6^ cells/mL in EGM2 MV containing 5% FBS, and then 7 µL resuspended cells were seeded into the left lane of the AIM Biotech 3D cell culture chip containing MP41 mCherry cells. A serum-free RPMI medium was added to the right lane of the chip to establish a serum gradient. The cell transmigration was observed daily with a fluorescence microscope. For testing KAI or control peptide, UM cells without inhibitor were mixed with collagen gel and put in the middle lane, KAI or control peptide (10 µM) were added to the left lane where hREC formed a barrio on the surface of the collagen gel.

Uveal melanoma metastasis mouse model

All animal experiments were carried out in compliance with the relevant laws and institutional guidelines and were approved by the Animal Care Committees administered through the Office of Animal Care and Institutional Biosafety at the University of Illinois at Chicago. Female SCID mice (4 weeks old, The Jackson Laboratory; 001803) were anesthetized intraperitoneally with ketamine/xylazine at 100 mg/kg and 8 mg/kg, respectively. 1% Tropicamide (Bausch Lomb, Laval, Canada) was administered to dilate the eyes for improved visualization. Following this, 0.5% Proparacaine (SANDOZ, Basel, Switzerland) was used to anesthetize the eyes, and Goniovisc eye lubricant (Hub Pharmaceuticals, Scottsdale, AZ, USA) was applied to maintain moisture. A precise incision was carefully made in the cornea, creating a small opening. A hole was made by a 30G needle prior to injection. A total of 3x10^5^ MP41 cells expressing mCherry and luciferase in 3 µl PBS were loaded into a Hamilton syringe equipped with a needle. These cells were gently introduced into the suprachoroidal space as described (PMID: 10711634). A tissue adhesive solution was employed to reseal the cornea, restoring its original state before the incision. Subsequently, 0.5% Erythromycin (Eye Supply, Tampa, FL) was administered to prevent any potential infections. Mice were placed on a heated pad to facilitate recovery. Mice were given a period of 7 days to develop tumors. For post-injection monitoring, mice were observed for changes in weight, eye size, and appearance. mCherry signal and Optical Coherence Tomography (OCT) were captured using the Phoenix MICRON OCT2 system from Phoenix MICRON (Bend, OR, USA). Injected MP41 was confirmed by its fluorescence and highly reflective signal in OCT. To track bioluminescence signals, an intraperitoneal injection of 150 mg/kg b.w. luciferin was administered, and bioluminescence was quantified using the Lago X system from Spectral Instruments (Tucson, AZ, USA) within 20 minutes of luciferin injection. All mice which received injection developed primary tumor in the suprachoroidal space 7 days post injection. Mice were divided into five groups to ensure a similar distribution of tumor burden across each treatment group and control group. Subsequently, they received daily treatments of either KAI (5 µg/eye) or a scramble peptide (5 µg/eye). Alternatively, another set of mice underwent intravitreal injection with either 2 µg Aflibercept or a vehicle on day 8.

Quantitative real-time PCR (qPCR) to detect circulating tumor cells (CTC)

8 weeks post-injection of UM cells, 100 μL mouse blood was collected by cheek puncture using animal lancets and collected to K2-EDTA tube (BD, Franklin Lakes, NJ, USA). 5 volumes of ammonium chlorides solution (150 mM NH_4_Cl, 0.1 mM EDTA buffered with KHCO_3_, pH7.2~7.6) (STEMCELL Technologies, Vancouver, Canada) was added to lyse red blood cells, and incubated on ice for 15 min. Cells were centrifuged at 1200 xg for 10 min at 4^o^C. Cell pellets containing mouse blood cells and human CTC were washed with PBS and centrifuged again at 1200 xg for 10 min at 4^o^C. Cells were suspended in 180 μL PBS. DNA was isolated from cell suspension using QIAamp DNA mini Blood kit (QIAGEN, Hilden, Germany), according to the manufacturer's procedure. Eluted DNA was tested for real-time PCR reactions. Primers are specific to humans (chromosome 17: forward 5’-GGG ATA ATT TCA GCT GAC TAA ACA G-3', reverse 5'- AAA CGT CCA CTT GCA GAT TCT AG -3') and mice (GAPDH: forward 5'- ACT CCA CTC ACG GCA AAT TC-3', reverse 5'- TCT CCA TGG TGG TGA AGA CA-3'). SYBR Green master mix was employed in a 96-well plate following the recommended cycling conditions. Data analysis involved the use of QuantStudio Design and Analysis Software to determine cycle threshold (Ct) values. The standard curve was drawn with known numbers of MP41 cells mixed with mouse blood. The number of CTCs was calculated based on the Ct values that fit the standard curve. Tumor-free mice were used as negative control. The average number of CTCs detected in tumor-free mice was considered as background noise. With this threshold, we determined CTC-positive or negative mice in tumor-bearing groups.

Statistical analysis

Data were analyzed with GraphPad Prism 10 (GraphPad, Sandiego, CA, USA). Two samples were compared using the Student's *t*-test. More than 2 samples were analyzed by one-way analysis of variance (ANOVA), followed by post hoc comparisons. Figure 1d was tested by Chi-squared test.
